# Detection of *Mycoplasma pneumoniae* in Simulated and True Clinical Throat Swab Specimens by Nanorod Array-Surface-Enhanced Raman Spectroscopy

**DOI:** 10.1371/journal.pone.0013633

**Published:** 2010-10-26

**Authors:** Suzanne L. Hennigan, Jeremy D. Driskell, Richard A. Dluhy, Yiping Zhao, Ralph A. Tripp, Ken B. Waites, Duncan C. Krause

**Affiliations:** 1 Department of Microbiology, University of Georgia, Athens, Georgia, United States of America; 2 Department of Infectious Diseases, University of Georgia, Athens, Georgia, United States of America; 3 Department of Chemistry, University of Georgia, Athens, Georgia, United States of America; 4 Department of Physics and Astronomy, University of Georgia, Athens, Georgia, United States of America; 5 Departments of Pathology and Microbiology, University of Alabama at Birmingham, Birmingham, Alabama, United States of America; Institut de Pharmacologie et de Biologie Structurale, France

## Abstract

The prokaryote *Mycoplasma pneumoniae* is a major cause of respiratory disease in humans, accounting for 20% of all community-acquired pneumonia and the leading cause of pneumonia in older children and young adults. The limitations of existing options for mycoplasma diagnosis highlight a critical need for a new detection platform with high sensitivity, specificity, and expediency. Here we evaluated silver nanorod arrays (NA) as a biosensing platform for detection and differentiation of *M. pneumoniae* in culture and in spiked and true clinical throat swab samples by surface-enhanced Raman spectroscopy (SERS). Three *M. pneumoniae* strains were reproducibly differentiated by NA-SERS with 95%–100% specificity and 94–100% sensitivity, and with a lower detection limit exceeding standard PCR. Analysis of throat swab samples spiked with *M. pneumoniae* yielded detection in a complex, clinically relevant background with >90% accuracy and high sensitivity. In addition, NA-SERS correctly classified with >97% accuracy, ten true clinical throat swab samples previously established by real-time PCR and culture to be positive or negative for *M. pneumoniae*. Our findings suggest that the unique biochemical specificity of Raman spectroscopy, combined with reproducible spectral enhancement by silver NA, holds great promise as a superior platform for rapid and sensitive detection and identification of *M. pneumoniae*, with potential for point-of-care application.

## Introduction

Mycoplasmas are small, cell wall-less prokaryotes having a minimal genome and limited biosynthetic capabilities that necessitate a symbiotic lifestyle. Some species constitute part of their host's normal flora, but pathogenic mycoplasmas are common and a significant threat to human and animal health. *Mycoplasma pneumoniae* is a major cause of respiratory disease in humans. Infections are acquired through respiratory secretions and manifest in nonspecific upper respiratory tract symptoms which may progress to tracheobronchitis and atypical bronchopneumonia. *M. pneumoniae* accounts for 20% of all community-acquired pneumonia and is the leading cause of pneumonia in older children and young adults [1]. However, the complexity of *M. pneumoniae* pathogenesis, which can include extrapulmonary spread, chronic sequelae such as asthma and COPD, and the potential for secondary infection, complicates diagnostic strategies.

Serologic testing has long been the foundation for diagnosis of *M. pneumoniae* infections, due to the considerable challenges posed by direct culture [2], [3]. Enzyme-linked immunoassay is the most widely used commercially available serologic test for M. *pneumonia*e in the U.S. and under ideal circumstances can yield comparable sensitivity to PCR [4]. However, this requires sufficient time following infection for an antibody response to develop and the availability of paired acute- and convalescent-phase sera, thereby limiting its value, particularly for point-of-care testing. PCR can exhibit high sensitivity and yield positive detection sooner than serological testing but is limited by issues of reliability, standardization, and cost [1], [5]. The inability to provide rapid and definitive diagnosis of *M. pneumoniae* infections delays initiation of appropriate treatment, prolongs morbidity, and increases the likelihood of continued transmission and long-term sequelae, highlighting the critical need for a new diagnostic platform with high sensitivity, specificity, and expediency.

The unique biochemical specificity inherent to vibrational spectroscopy has led to its evaluation for detection and identification of infectious agents. Raman spectroscopy has several features appropriate for biological samples, including narrow bandwidths, good spatial resolution, and applicability to aqueous samples, but the inherently low scattering cross-section of standard Raman spectra has prevented its more widespread application for biosensing purposes. The enhancement of the Raman spectrum of an analyte in proximity to metal surfaces (surface-enhanced Raman spectroscopy, or SERS) can significantly increase spectral intensity without loss of specificity, and consequently molecular fingerprinting based on SERS has been applied to microorganisms with considerable success [6]–[8]. However, realization of the potential of SERS for biosensing applications has been limited by inconsistency in the reproducibility and preparation of SERS-active metal substrates, requiring optimized nanofabrication strategies. To this end we have shown that oblique angle vapor deposition can reproducibly yield aligned high aspect ratio silver nanorod array (NA) substrates that generate SERS enhancement factors of >10^8^ and <15% batch variation [9]. A second critical element in the use of vibrational spectroscopy as a diagnostic tool is the use of algorithms for specialized feature selection, rather than examination of individual peaks. Chemometric analysis reduces the dimensionality of the dataset, maximizes the variance among spectral fingerprints, and provides a measure of the reproducibility and sensitivity of the spectroscopic method. The silver NA-SERS platform described here was previously shown to exhibit outstanding sensitivity and specificity for detection and differentiation of closely related strains of respiratory syncytial virus and rotavirus, even in biochemically complex backgrounds [10], [11]. In the current study we demonstrated the capacity of this biosensing platform to detect and distinguish with exceptional sensitivity, both closely and more distantly related *M. pneumoniae* strains in culture, and to detect *M. pneumoniae* in simulated throat swab samples, representing a biochemically complex and clinically relevant background. Furthermore, we analyzed ten true clinical throat swab samples previously determined to be positive or negative for *M. pneumoniae* by both real-time PCR and culture, correctly classifying these spectra by NA-SERS with >97% accuracy, and providing an indication of the potential this technology may have for clinical application.

## Methods

### Mycoplasma strains and sample preparation

Wild-type *M. pneumoniae* strains M129 and FH were used in this study, representing two major *M. pneumoniae* subtypes which shift in dominance over a 4–7-year periodicity [12], [13]. Despite reports of new subtypes, there is good support for examination of these two populations [13], [14]. We also included strain II-3, a spontaneously-arising avirulent mutant derived from M129 [15], [16]. Mycoplasmas were cultured in SP4 medium [17] in tissue culture flasks at 37°C and harvested when the phenol red pH indicator was orange (pH approx. 6.5), indicating log phase growth. For strains M129 and FH the spent growth medium was decanted and cells collected by scraping in HPLC grade water, centrifuged, and washed 3X. For strain II-3, which fails to attach to plastic (15), cell suspensions were collected by centrifugation and likewise washed 3X. Washed mycoplasmas were suspended in 500 µl water, syringe-passaged 10x with a 25-gauge needle, and aliquots of each were serially diluted in water and plated on PPLO agar [18] for colony-forming unit (CFU) determination. Water rather than buffered saline was used initially to avoid possible damage to the silver nanorods by chloride ions, and therefore parallel samples were also processed in 10 mM phosphate-buffered saline (PBS, pH 7.2) to assess loss of viability in water. Mycoplasmas were fixed in 4% formalin for 3 h and inactivation confirmed by 3-wk incubation in SP4 medium at 37°C with no change in the phenol red indicator. In subsequent studies samples harvested in PBS or SP4 medium and formalin-fixed before dilution with water exhibited no loss of NA-SERS sensitivity or specificity or evidence of damage to the NA (data not shown).

Throat swabs were collected from two of us, for which Institutional Review Board (IRB) approval was not sought, as the procedure was non-invasive, there were no risks involved, no identifiers were included on the samples, and no additional participants were recruited. The University of Georgia IRB reviewed and approved the studies using banked true clinical samples. For simulated infection studies, samples were collected using sterile, polyester-tipped swabs (Puritan Medical Products Co, Guilford, ME) and extracted with 1 ml HPLC grade water by swirling and expressing against the side of the tube, syringe-passaged for cell dispersion, and aliquoted for further processing. Cultures of each in SP4 were negative for *M. pneumoniae* growth after 6-wk incubation. Some aliquots were subsequently spiked with serial 10-fold dilutions of fresh *M. pneumoniae* culture in water, and each sample was fixed with 4% formalin. For analysis of true clinical samples we used pre-existing banked throat swab samples previously collected at the University of Alabama at Birmingham (UAB) using UAB IRB-approved protocols. Samples were extracted in SP4 medium containing polymyxin B, ampicillin, and nystatin (500 units/ml, 1 mg/ml, 50 units/ml, respectively), tested by culture and by real-time PCR [19], and stored frozen at −80°C. For NA-SERS testing, 3-µl aliquots were removed and diluted 1∶100 in water, with 1-µl volumes analyzed by NA-SERS in duplicate as described below.

### NA-SERS measurements and chemometrics analysis

Silver NA substrates were prepared for reproducible enhancement of the Raman signal as described [11], [20], [21]. Briefly, an electron beam evaporation system deposited three sequential layers: a 20-nm Ti film, a 500-nm Ag film, and an obliquely angled Ag NA. We used individual 1×1 cm substrates or 1×3 inch substrates with 40 3-mm diameter polydimethylsiloxane-formed wells [22]. Average specifications for Ag nanorods for optimum signal production were previously determined to be approximately 900-nm length, 90–100-nm diameter, 13 nanorods/µm^2^ surface density, and formed 71° with respect to the surface normal [21]. Surface-enhanced Raman spectra were acquired using a Renishaw inVia Reflex multi-wavelength confocal imaging microscope (Hoffman Estates, IL). A Leica apochromatic 5× objective (NA 0.12) illuminated a 1265 µm^2^ area on the substrate, allowing spatial averaging and minimizing the effect of potential random hot spots. A 785-nm near-infrared diode laser (Renishaw) operating at 5% power capacity (15 mW) provided the incoming radiation, with spectra collected in 10-sec acquisitions. Sample imaging was accomplished by line-focus optics (diffraction grating of 1200 lines/mm). Mycoplasma samples in 1 µl volumes were applied to nanorod array substrates, and spectra from up to 10 random locations on each were analyzed to assess spectral variation within a single substrate and among different substrates. Raman spectra between 400–1800 cm^−1^ were collected using Renishaw WiRE2.0 software. Instrument settings were optimized to maximize signal and minimize saturation or degradation of samples by the laser. Sample dilution, particularly with throat swab specimens, eliminated formation of a visible film on the substrate, which can prevent direct contact by the analyte and diminish sensitivity, as we have described previously [11].

Raman spectra were first averaged using GRAMS32/A1 spectral software package (Galactic Industries, Nashua, NH) to assess signal-to-noise quality, and baseline-corrected using a concave rubberband algorithm which performed 10 iterations on 64 points to aid in preliminary evaluation and peak assignment (OPUS, Bruker Optics, Inc., Billerica, MA). Raw spectra were pre-processed for chemometrics using the first derivative of each spectrum and a nine-point, 2nd-order polynomial Savitsky-Golay algorithm [23] followed by normalization to unit vector length with Unscrambler (CAMO, Freiberg, Germany) or Matlab 7.8 (Natlick, MA) software to allow direct intensity comparison between samples and between variables within a sample [24]. The unsupervised methods of principal component analysis (PCA) and hierarchical cluster analysis (HCA) were used to explore clustering of similar spectra using Unscrambler software [24], [25]. The diagonal matrix (y-block) for partial least squares discriminatory analysis (PLS-DA) was generated in Excel and imported into PLS toolbox 4.0 (Eigenvector, Wenatchee, WA) operating in Matlab [26]. Both *x-* and *y-* block data were mean-centered prior to PLS-DA [27]. Model robustness was tested by cross-validation (CV) with Venetian Blinds, which builds a classification model based on 90% of the data, tests the remaining 10% against that model, and repeats the process through ten iterations to assess its predictive power.

### PCR analysis

DNA from 200 µl of each serial dilution was processed with the QIAamp DNA Blood Mini Kit (QIAGEN, Valencia, CA) as recommended, except that the final centrifugation of the eluate (50 µl) was performed after a 5-min elution. PCR was carried out with a 16S rRNA primer set specific for *M. pneumoniae* [28] using the EasyStart™ 100 PCR mix-in-a-tube kit (Molecular Bio-Products, San Diego, CA). Briefly, 5 µl of DNA, 0.31 µM each forward and reverse primer, water, and 1 µl TaqPol (Fischer BioTech, Pittsburgh,PA) were combined to a final volume of 50 µl. Reactions were carried out using an Eppendorf Mastercycler (Hamburg, Germany) with thermocycler settings of 94°C for 2 min for cycle 1; 40 cycles of denaturation and extension at 94°C for 1 min, 55°C for 1 min, 72°C for 2 min for cycle 2; and hold indefinitely at 4°C for cycle 3. 10-µl volumes were analyzed by agarose gel electrophoresis and ethidium bromide staining for the expected 277-bp product.

## Results and Discussion

### Differentiation of *M. pneumoniae* strains

**Figure 1:**
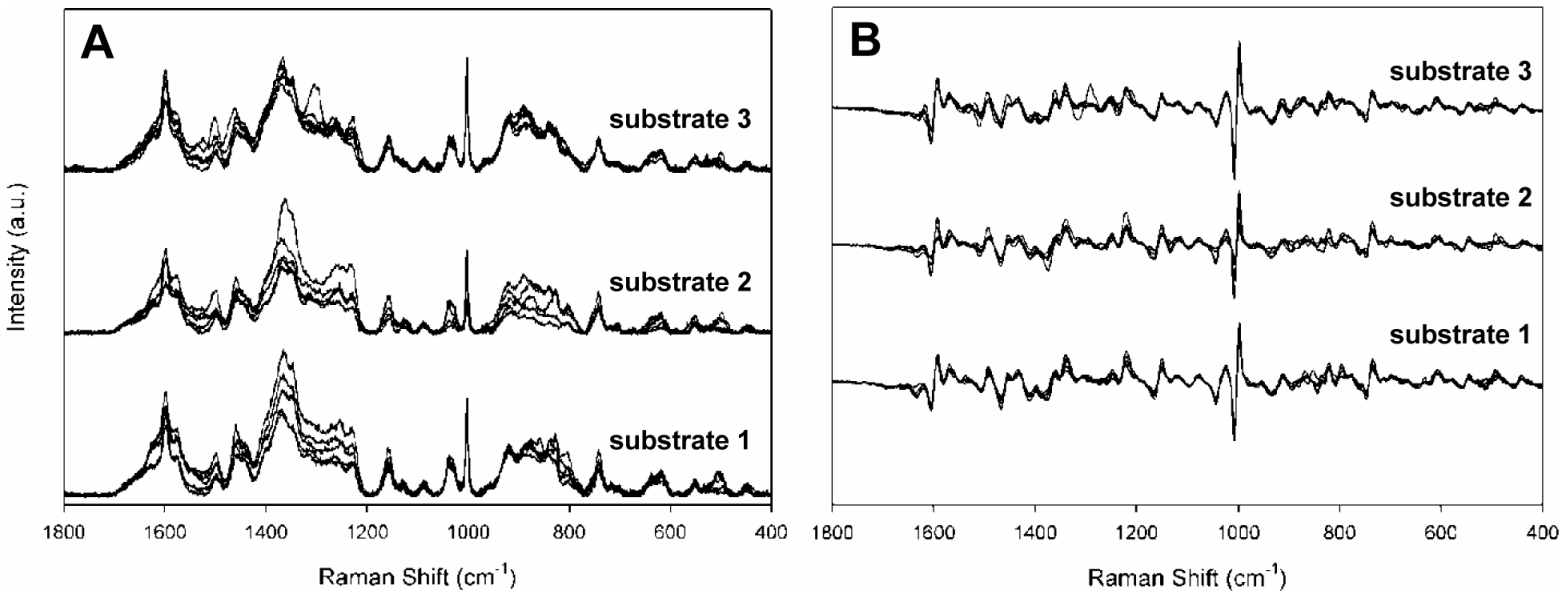
Reproducibility of spectra. Raw spectra (**A**) of *M. pneumoniae* strain FH collected from five random spots from three separate NA substrate wells, baseline corrected and offset. (**B**) First derivative spectra for strain FH from Figure 1A.

**Figure 2:**
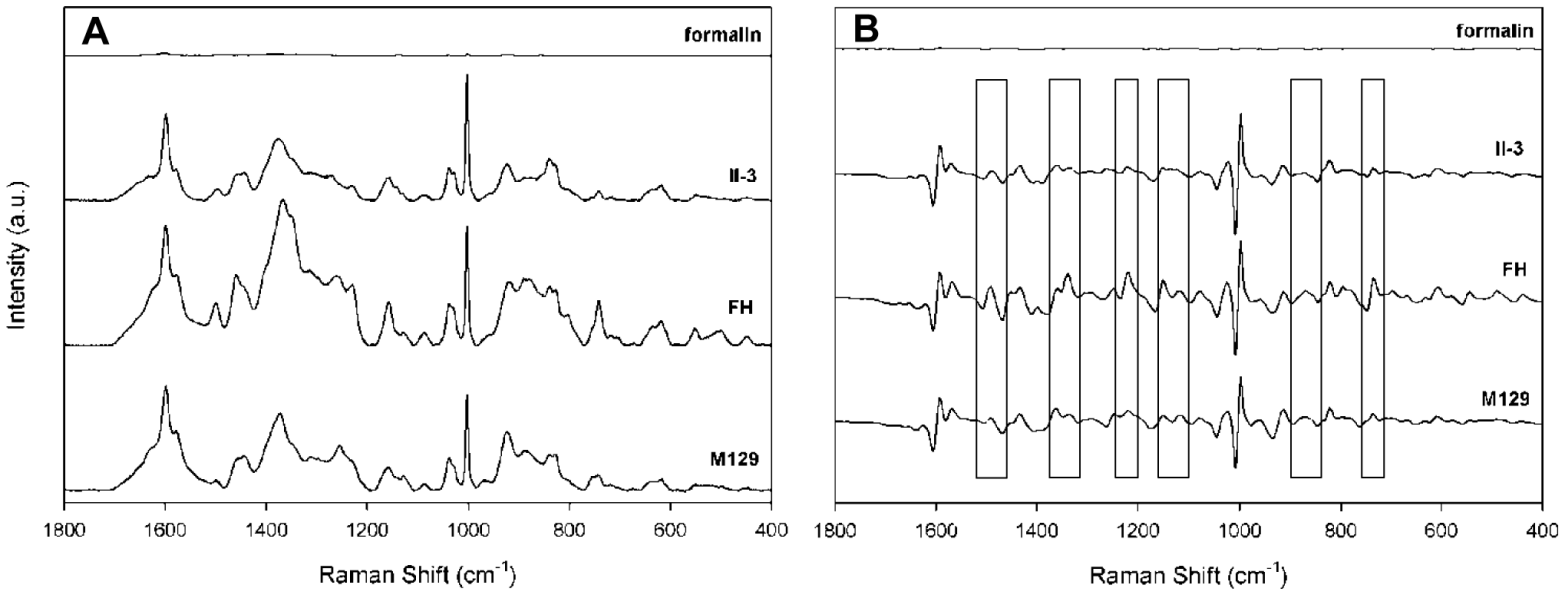
Differentiation of *M. pneumoniae* strains. (**A**) Average spectra (n = 15) of *M. pneumoniae* strains with formalin background, baseline corrected and offset; and (**B**) first derivative spectra from panel A demonstrating strong similarities but also clear differences (boxes) among the strains.

We compared baseline-corrected raw spectra for *M. pneumoniae* strains FH, M129, and II-3 to assess reproducibility, collecting five spectra from three separate wells on a 40-well array or three independently fabricated substrates (Figure 1A and data not shown), for a total of 15 spectra per strain. Alignment of the spectra established their similarity with respect to Raman band location and intensity but also revealed variation with spot and substrate. To improve resolution of overlapping bands and eliminate artifacts potentially introduced by baseline correction we also compared first derivative spectra of these samples (Figure 1B and data not shown). Comparison of the averaged spectra and first derivatives thereof for all three strains collected from different substrates (Figures 2A and 2B, respectively) established strain similarity in Raman band wave number and intensity but also revealed consistent strain differences, most notably bands at 1460, 852, and 785 cm^−1^, and the regions between 1325–1375, 1230–1295, and 1125–1175 cm^−1^ (Figure 2B, boxes), underscoring the need for multivariate statistical analysis for further differentiation. The background spectrum for formalin was inconsequential.

**Figure 3:**
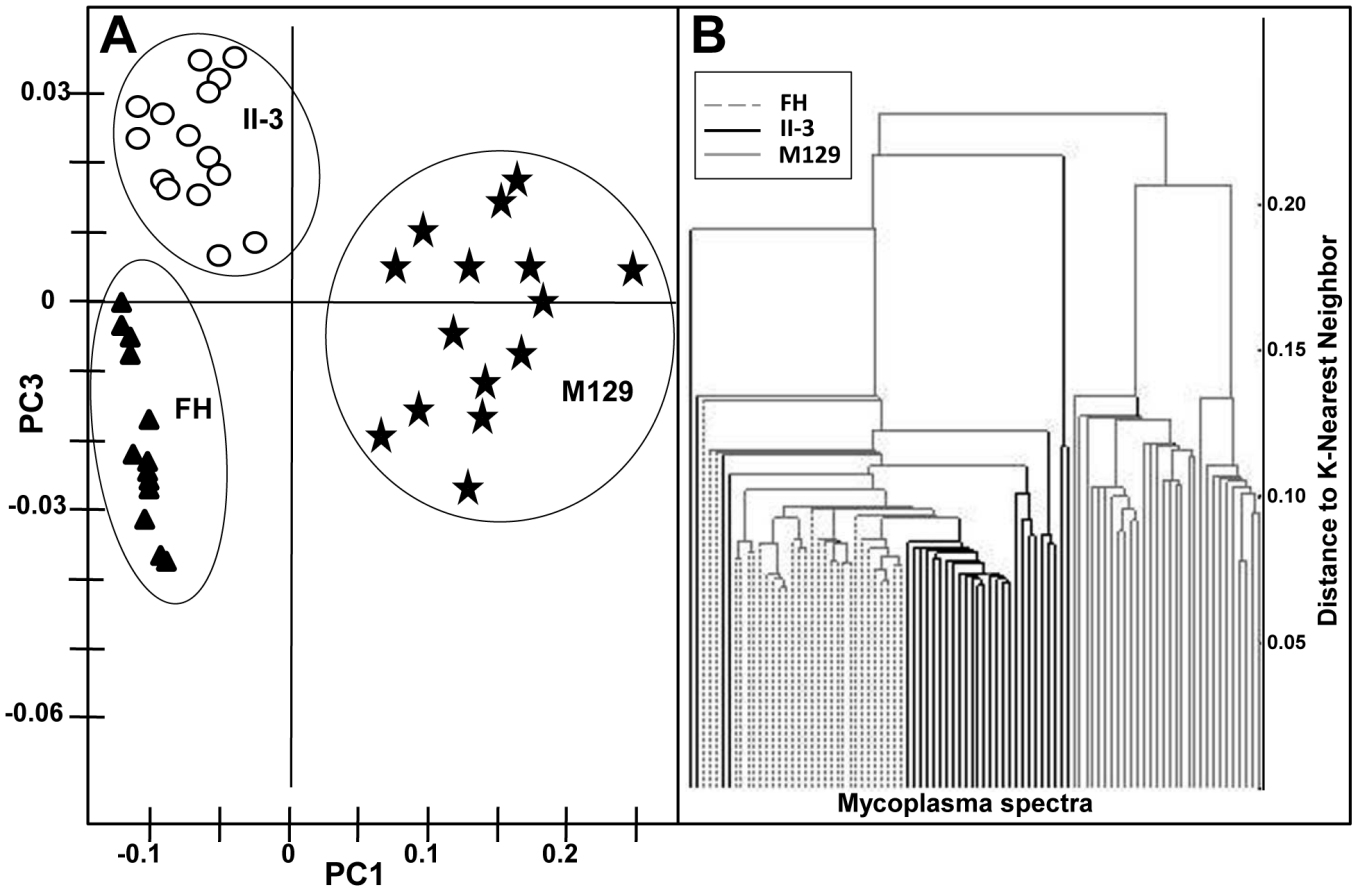
Principal Component and Hierarchal Cluster Analyses. Chemometric analysis was conducted on the spectral data from Figure 2. (**A**) PC scores plot 1vs. 3 of *M. pneumoniae* strains FH, M129, and II-3, as indicated. 77% of the variance was captured in PC1 and 3% in PC3 to distinguish the strains. (**B**) HCA of pre-processed spectra of *M. pneumoniae* strains FH (dashed lines), M129 (solid lines), and II-3 (bold lines). Four spectra from strain II-3 were misclassified with FH (at left).

PCA and HCA are unsupervised statistical methods for data analysis and form classes in the context of total variance [29], [30]. PCA reduces dataset dimensionality and facilitates establishing patterns and grouping of similar spectra for classification. Though limited by 2-D projection, PCA by design explains successively smaller proportions of the variance, with the first few principal components explaining the greatest percentage of total variance. Comparison of processed spectra principal components 1 and 3 clearly differentiated the three strains (Figure 3A), where each data point corresponds to a single SERS spectrum. HCA is considered more robust in the generation of classes than PCA due to inclusion of additional dimensions [25], [31] and correctly grouped 86 of 90 samples, with only 4 spectra from II-3 improperly clustered with FH (Figure 3B).

PLS-DA is a full-spectrum, multivariate, supervised method whereby prior knowledge of the classes (i.e. mycoplasma strains) is used to yield more robust discrimination [27], [32], minimizing variation within classes while emphasizing latent variables between or among classes. PLS-DA was applied to establish the statistical significance of differences between the SERS spectra of the mycoplasma strains. PLS-DA generated a model from 90 spectra (30 per strain; same spectra as in Figure 3) with four latent variables accounting for 57% of the total variance, differentiating the strains with 93–100% sensitivity and specificity (Table 1); a CV error <0.05 was considered statistically insignificant as a diagnostic threshold [33], [34].

**Table 1:** PLS-DA of NA-SERS specificity and sensitivity in discriminating *M. pneumoniae* strains.

Modeled class^a^	II-3	FH	M129
Sensitivity (CV^b^)	0.933	1.000	1.000
Specificity (CV)	1.000	1.000	1.000
Class Error(CV)	0.03	0	0
RMSEC^c^	0.066	0.087	0.043

a Four latent variables, accounting for 57% of the total variance, were used to generate the model.

b CV, cross validation.

c RMSEC, root mean squared error in calibration.

Key differences in several major surface proteins have been described for *M. pneumoniae* strains M129 and FH [35]–[37], hence the capacity of NA-SERS to distinguish these was not surprising based on recent success using this platform to distinguish rotavirus strains [11]. However, strain II-3 is derived from M129, with a nucleotide deletion in MPN453 resulting in loss of adhesin protein P30 and reduced levels of adherence-associated protein P65 [15], [38], and therefore the capacity to distinguish M129 from II-3 was unexpected and underscores the discriminatory power of this technology. Protein P65 also differs between M129 and FH [36] and may represent a common denominator in the distinct spectral signatures of these strains.

### Sensitivity and limits of detection

**Figure 4:**
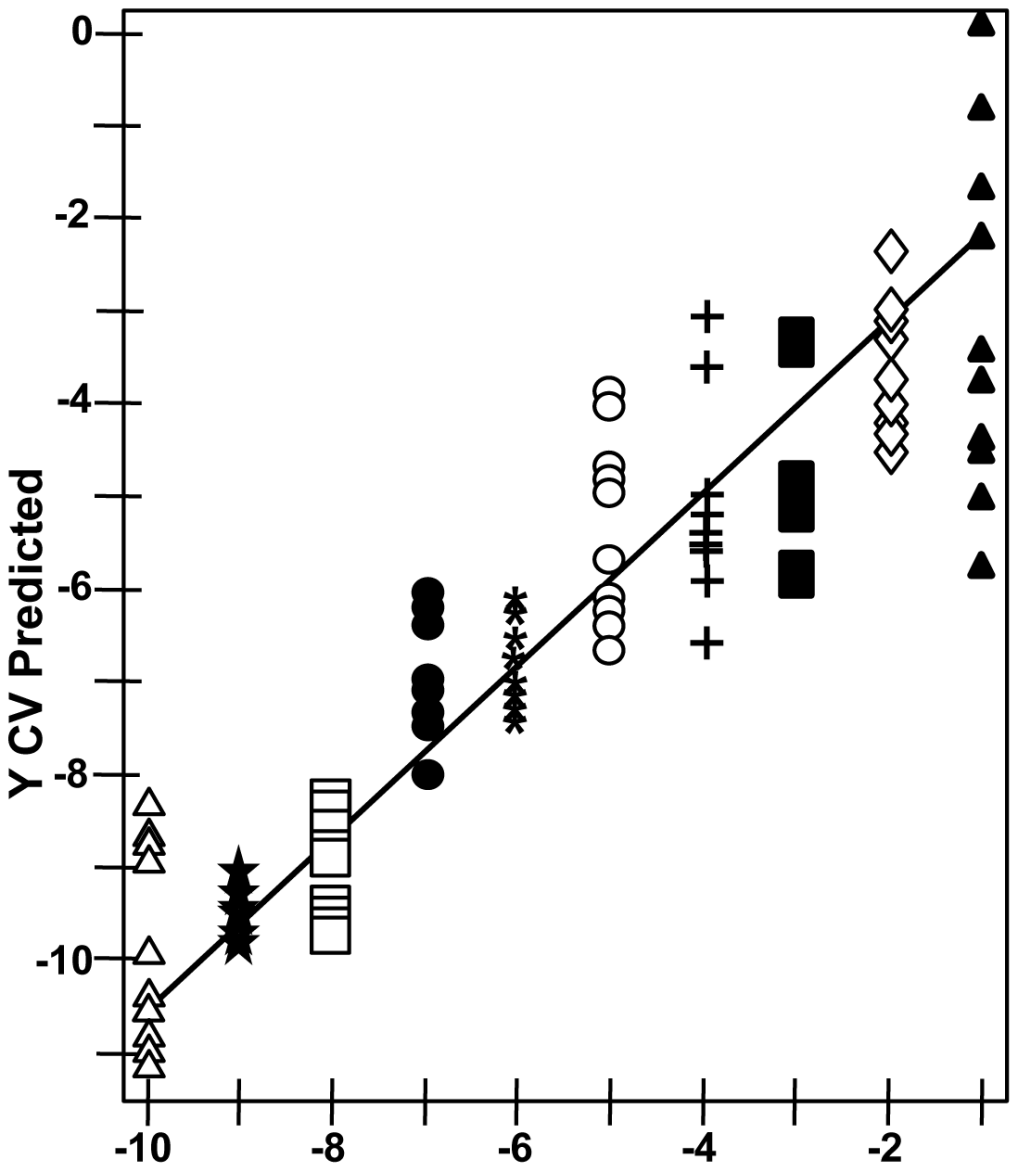
PLS regression analysis of serial dilutions of *M. pneumoniae.* Serial 10-fold dilutions of strain II-3 spanning 10 logs of concentration were assessed by PLS regression for degree of linearity in actual intensity of the measured spectra plotted against the predicted intensity. Starting concentration, 1.8×10^9^ CFU/ml; R^2^ = 0.810.

**Figure 5:**
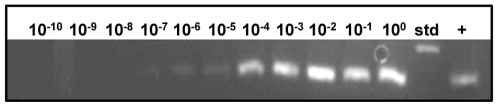
PCR analysis of serial dilutions of *M. pneumoniae* strain II-3. Dilutions 10^−10^ to 10^0^ are indicated; std, DNA size standard (350 kbp); +, positive control (277 kbp). Starting concentration, 1.8×10^9^ CFU/ml.

To assess NA-SERS sensitivity further we analyzed the spectra from ten serial ten-fold dilutions (10^−1^ to 10^−10^) per strain by PLS-DA in a single model (Table 2), detecting and differentiating the three strains with >94% sensitivity and specificity, an outcome especially meaningful given the range in bacterial loads likely in clinical samples. Spectra from the serial dilutions of strain II-3 were also assessed by PLS regression analysis, revealing a correlation between true mycoplasma concentration and that predicted for cross-validated samples (Figure 4). This correlation suggests that spectral intensity reflects concentration, although signal quenching was apparent with the most concentrated mycoplasma suspensions. High SERS enhancement is achieved through the local electric field between nanorods and requires that the sample penetrate within the array itself [39], likely accounting for the quenching effect at high sample concentrations. A similar linear relationship was observed in the quantitative detection of rotavirus by NA-SERS [11]. Finally, *M. pneumoniae* strain II-3 spectra were correctly differentiated at dilutions as high as 10^−9^, corresponding to a lower limit of detection of 0.02 CFU/sample analyzed on the substrate; a comparable limit was observed with strain M129 (data not shown). By comparison, standard PCR performed on parallel samples yielded the expected 277-bp product from primers specific for the *M. pneumoniae* 16S rRNA gene through the 10^−7^ dilution (Figure 5), which corresponds to a lower detection limit by standard PCR of 3.6 CFU/reaction, or 0.7 CFU analyzed on the gel, consistent with the range reported for *M. pneumoniae* by diagnostic PCR [40]. It is difficult to extrapolate these results to the single-cell level for mycoplasmas, which tend to clump and for which CFU and plating efficiency are poorly defined [3]. Furthermore, we also cannot rule out the possibility that intact cells are not essential, and the ability to detect *M. pneumoniae* spectra may fall below the single-cell level due to recognition by SERS of cell components in cell lysates, as was reported recently for mycobacteria [41].

**Table 2:** PLS-DA of NA-SERS specificity and sensitivity in discriminating three *M. pneumoniae* strains and two negative controls (formalin and water).

Modeled class^a^	formalin	II-3	FH	M129	water
Sensitivity (CV)	0.98	0.96	0.941	1.000	1.000
Specificity (CV)	0.981	0.955	0.955	0.987	1.000
Class error (CV)	0	0.04	0	0.006	0
RMSEC	0.08	0.15	0.17	0.06	0.05

a abbreviations the same as in Table 1; single model generated using 12 latent variables accounting for 88% of the total variance for all serial dilutions of each strain.

### Analysis of simulated and true clinical throat swab samples

**Figure 6:**
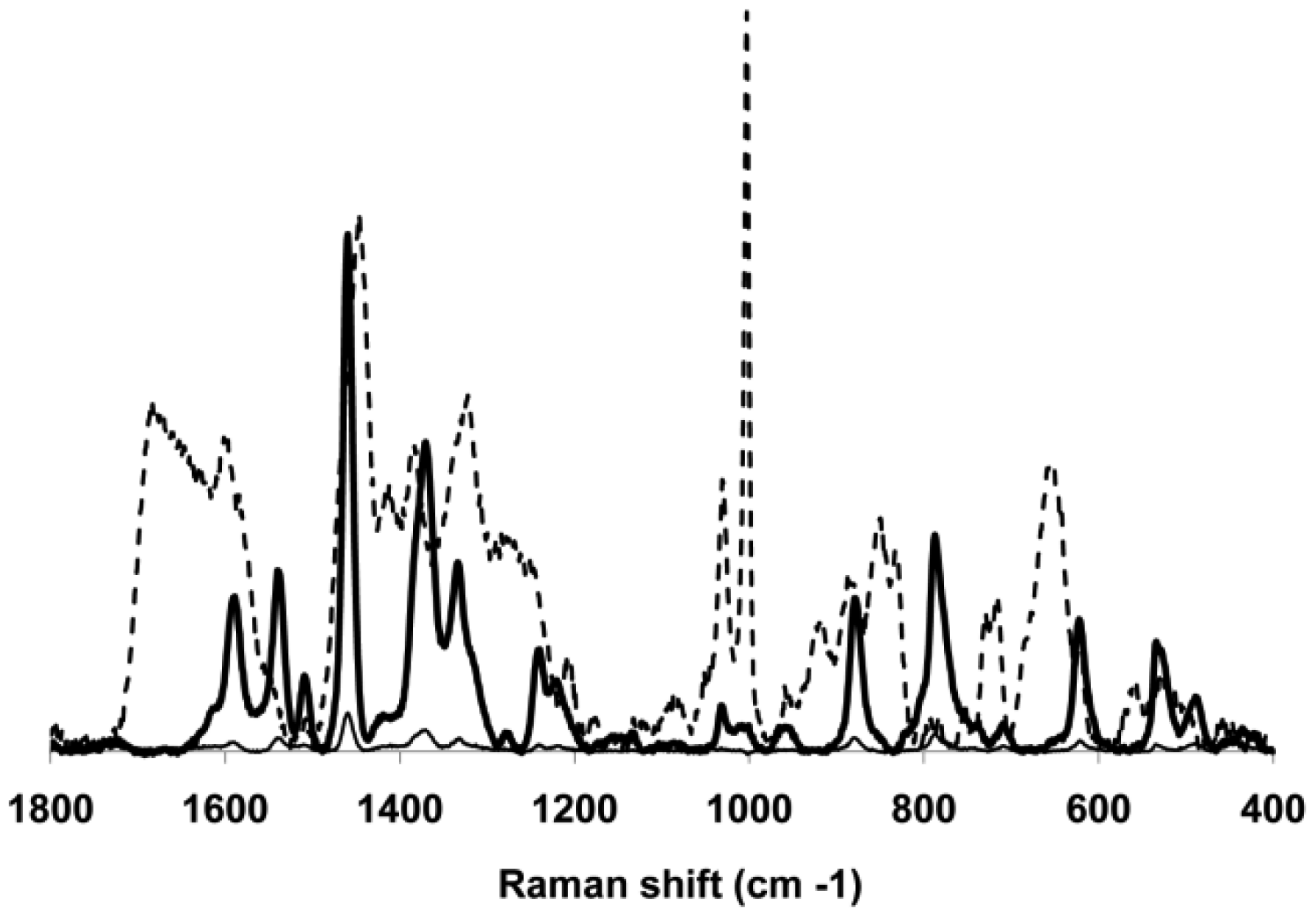
Differentiation of spiked and control throat swab samples. Representative difference spectra of spiked, pooled throat swab samples after subtraction of the spectrum of the un-spiked, pooled throat swab control. Bold and thin solid lines, spiked samples (8.2×10^4^ and 8.2×10^3^ CFU *M. pneumoniae* M129/µl, respectively); dashed line, representative spectrum of *M. pneumoniae* M129.

**Figure 7:**
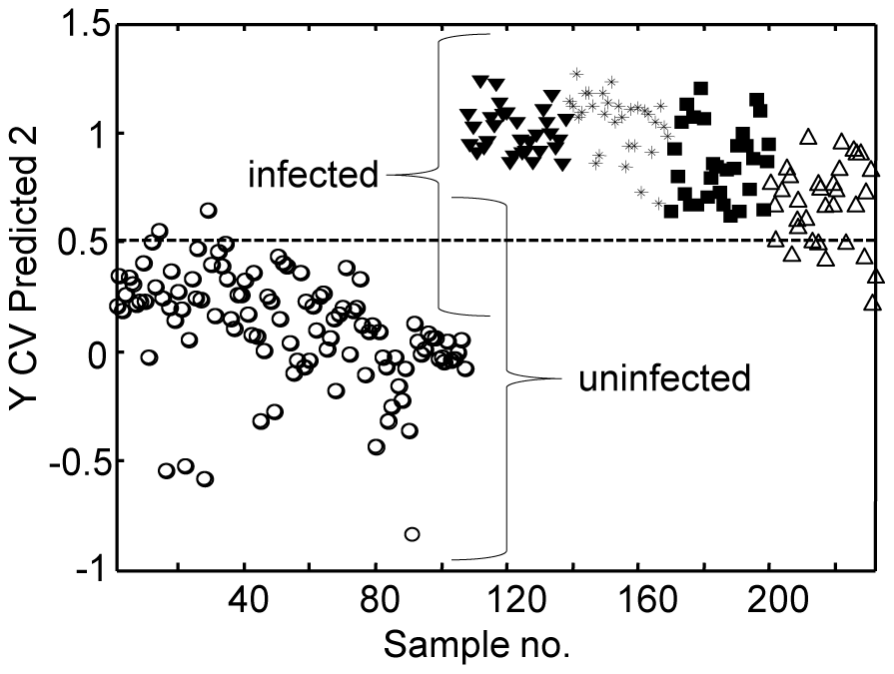
PLS-DA of pooled throat swabs spiked with known concentrations of *M. pneumoniae* strain M129. Open circles, uninfected pooled throat swab samples; solid triangles, spiked throat swab samples with 8.2×10^4^ CFU/µl; asterisks, spiked throat swab samples with 8.2×10^3^ CFU/µl; squares, spiked throat swab samples with 8.2×10^2^ CFU/µl; open triangles, spiked throat swab samples with 8.2×10^1^ CFU/µl. A downward trend across the spiked samples toward the threshold (dotted line) suggests that the model makes more errors in classification with the higher dilution factors.

**Figure 8:**
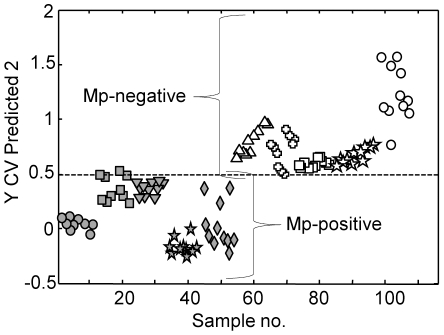
PLS-DA of true clinical throat swab samples from different individuals. Gray symbols, samples previously shown to be *M. pneumoniae-*negative (Mp-negative) by culture and real-time PCR from five persons; open symbols, samples previously shown to be *M. pneumoniae-*positive (Mp-positive) by culture and real-time PCR from five persons. Five or six spectra were collected for each sample, in duplicate.

We evaluated NA-SERS for the detection of *M. pneumoniae* M129 in simulated throat swab samples as an initial assessment of its performance in a biochemically complex background. We analyzed spectra collected from individual and pooled throat swab samples spiked with *M. pneumoniae* against control throat swab samples (ten replicates of 1 µl each). Difference spectra from spiked, pooled throat swab samples (8.2×10^4^ and 8.2×10^3^ CFU/µl) after subtraction of the spectrum from the corresponding unspiked pooled throat swab sample (Figure 6), provided an indication of the contribution of *M. pneumoniae* to the spectral fingerprints of each sample. For comparison, the average spectrum of *M. pneumoniae* M129 is overlaid, revealing similar Raman band positions and intensities with the difference spectra. Particularly well aligned and correlating with the presence of *M. pneumoniae* were peaks at 1598, 1459, 1367, 1332, 1241, 1031, 1004, 882, 619 and 525 cm^−1^ (Figure 6), and for most their intensity correlated with *M. pneumoniae* CFU in the spiked samples (Figure 6 and data not shown). These bands have been characterized previously in bacterial samples [42]–[44], with peak alignment at 1598 cm^−1^ associated with a C = C lipid; at 1459 cm^−1^ with protein; at 1331 cm^−1^ with the C–H bend from protein; at 1241 cm^−1^ a well-established amide III band; at 1004 cm^−1^ characteristic of aromatic breathing modes in phe or tyr; at 882 cm^−1^ characteristic of C-C; and at 525 cm^−1^ attributed to C-S-S-C bonds characteristic of cys and sulfated sugars. The slight shifts in several of the bands can be explained by a matrix effect, i.e. vibrational contributions of each background. However, we are primarily interested in the entire spectral fingerprint for potential diagnostic applications and therefore used the multivariate PLS-DA to evaluate further the overall spectral signatures for each dilution. A model built with all dilutions and classification as positive or negative for *M. pneumoniae* indicated >90% accuracy in cross-validated analysis (Figure 7 and Table 3). The CV accuracy ranged from 0 to 0.105 when analyzed by individual dilution factor, while the full dataset of all dilutions yielded a CV error of 0.033, corresponding to 98.1% specificity and 95.2% sensitivity for mycoplasma detection in this biochemically complex, clinically relevant background. Similar results were obtained when throat swab samples were not pooled prior to spiking (data not shown). The lower detection limit here of 82 CFU is comparable to that reported for real-time PCR analysis of actual and spiked clinical samples [45] but may underestimate the true limits of detection for throat swab samples, as higher dilutions were not examined. Finally, as an initial assessment of the potential of NA-SERS for detection of *M. pneumoniae* in true clinical throat swab samples, we tested ten specimens (five of each) previously shown to be positive or negative by both real-time PCR and culture. Five or six spectra were collected for each sample, in duplicate, with PLS-DA correctly classifying the spectra for all ten specimens as positive or negative for *M. pneumoniae* with >97% accuracy (Figure 8 and data not shown).

**Table 3:** PLS-DA of throat swabs spiked with serial dilutions of *M. pneumoniae*.

Dilution (CFU/sample)	Captured variance (x/y)			RMSECV	CV error^a^	Sensitivity	Specificity
10^0^ (8.2×10^4^)		36/94	.103		0	1.000	1.000
10^−1^ (8.2×10^3^)		72/93	.166		0	1.000	1.000
10^−2^ (8.2×10^2^)		71/79	.244		.0206	.968	.991
10^−3^ (8.2×10^1^)		66/58	.329		.105	.906	.882
All dilutions		75/83	.252		.033	.952	.981

a abbreviations same as in Table 1.

### Conclusions

*M. pneumoniae* is a significant human respiratory tract pathogen with respect to both incidence and impact, but diagnostic strategies are complicated by symptoms that are typically nondescript, a complex disease presentation that can include extra-pulmonary sequelae, and multiple challenges posed by direct culture. Serologic testing is the current method of choice for diagnosis but suffers from severe limitations which make it impractical and unreliable, while PCR can exhibit high sensitivity but is prone to false negatives. Thus, the lack of an adequate diagnostic platform for rapid detection with high sensitivity, specificity, and expediency is a critical barrier to improved control of *M. pneumoniae* infections.

The development of novel biosensors is yielding promising new direct approaches for rapid and sensitive detection of infectious agents. We previously established that this silver NA-SERS platform exhibits robust and reproducible spectrum enhancement for biosensing applications for viruses in pure preparations and in cell lysates [10], [11]. Here we extended those findings to bacteria with a first assessment of detection of *M. pneumoniae* in culture, in spiked throat swabs, and in true clinical samples. The platform described here detected and differentiated three *M. pneumoniae* strains in culture with excellent specificity and sensitivity and a sub-CFU lower limit of detection. Furthermore, a lower limit of detection at least as low as 82 CFU per sample was observed with spiked throat swab samples, where a loss of sensitivity was anticipated given the increased background complexity, as previously observed with rotavirus detection by NA-SERS in cell lysates [11].

Several potential challenges exist to widespread clinical application of NA-SERS for mycoplasma detection. For example, the increased biochemical complexity and variability in clinical specimens could confound interpretation of the spectral patterns. A recent study with clinical isolates of *M. pneumoniae* concluded that standard Raman spectra (without surface enhancement) were not informative for diagnostic differentiation of virulent and avirulent strains [46]. However, a different study reports that SERS spectra of bacteria are less congested and exhibit greater species differentiation than their non-SERS counterparts [47]. This point is underscored by our findings that this NA-SERS platform can detect and distinguish viruses in cell lysates with high sensitivity [11] and differentiate *M. pneumoniae* at the strain level (this study). A comprehensive analysis of diverse *M. pneumoniae* clinical isolates is underway to assess further the discriminatory capacity of NA-SERS. It will also be necessary to expand the testing of spiked samples to include a broader population as background controls and to assess detection in alternative clinical samples such as throat washes, nasopharyngeal swabs, and sputa. Moreover, variability in normal flora, including other mycoplasma species, and in some cases the potential presence of secondary agents, will complicate spectral analysis and must be negotiated in discriminating an *M. pneumoniae* spectral signature. It is also recognized that simulated throat swab samples may not model true clinical samples accurately. In particular, the background will likely reflect upper respiratory tract inflammation in true clinical samples, while the interplay between mycoplasma and host may differ with respect to colonization parameters. However, our data with true *M. pneumoniae*-positive and –negative clinical samples reveal comparable sensitivity and specificity to results with simulated infection (Figure 8). Finally, the potential impact of over-fitting a statistical model must be considered, although the use here of independent validation to assess the prediction capacity of our model yielded promising results (data not shown). Nevertheless, taken together, our experimental findings demonstrate the potential of this NA-SERS diagnostic platform for expedient detection and identification of *M. pneumoniae* in clinical samples with high sensitivity and specificity.
